# AIM2 Stimulation Impairs Reendothelialization and Promotes the Development of Atherosclerosis in Mice

**DOI:** 10.3389/fcvm.2020.582482

**Published:** 2020-11-11

**Authors:** Enzo Lüsebrink, Philip Roger Goody, Catharina Lahrmann, Anna Flender, Sven Thomas Niepmann, Andreas Zietzer, Christian Schulz, Steffen Massberg, Felix Jansen, Georg Nickenig, Sebastian Zimmer, Alexander Otto Krogmann

**Affiliations:** ^1^Medizinische Klinik und Poliklinik I, Klinikum der Universität München, Munich, Germany; ^2^DZHK (German Center for Cardiovascular Research), Partner Site Munich Heart Alliance, Medizinische Klinik und Poliklinik I, Klinikum der Universität München, Munich, Germany; ^3^Department of Internal Medicine II, Heart Center Bonn, University Hospital Bonn, Bonn, Germany

**Keywords:** Innate immune system, AIM2, atherosclerosis, immune system, mice

## Abstract

**Background:** Atherosclerosis has been shown to result from chronic inflammation caused by constitutive activation of the pattern recognition receptors (PRR), which are principle effectors of the innate immune system. PRR are present in the endosome or on the cellular membrane and can sense the aberrant release of nucleic acids, which is often a sign of acute or chronic cellular damage. Absent in melanoma 2 (AIM2) is a PRR that is expressed by vascular cells and specializes in detecting cytoplasmic double-stranded DNA (dsDNA). Activation of AIM2 leads eventually to activation of the inflammasome, but the role of AIM2 in vascular disease and atherosclerosis has not been well-studied. Therefore, in this study we took advantage of acute and chronic models of vascular injury to determine the biological role of AIM2 in atherogenesis.

**Methods and Results:** We were able to induce significant release of proinflammatory cytokines in mice through the intravenous injection of a synthetic ligand for AIM2, double-stranded poly dA:dT. This cytokine release was shown to impair reendothelialization of the carotid artery and increase the number of circulating endothelial microparticles (EMP) after acute denudation, compared to treatment with vehicle. We saw an increase in the production of reactive oxygen species in the aorta, the number of circulating EMP, and, most interestingly, atherosclerotic plaque formation in apolipoprotein E-deficient (ApoE^−/−^) mice when they received continual subcutaneous poly dA:dT, in contrast to vehicle-treated animals. Finally, treatment with poly dA:dT did not impair vascular reendothelialization in AIM2^−/−^ mice compared to vehicle controls in the carotid artery injury model.

**Conclusion:** Overall, our data suggest that AIM2, as a known regulator of the inflammasome, is an active participant in atherogenesis, and highlight the importance of fully understanding the pathological mechanisms involved. It seems to be worth of further exploration as a therapeutic target, and future studies focusing on the effects of AIM2 activation as well as its pharmacological inhibition may reveal promising new therapeutic concepts for the treatment of atherosclerosis.

## Highlights

- Stimulation of AIM2 works in a pro-atherosclerotic manner to promote the disruption of endothelial function, higher production of ROS in the vessels, and a decrease in reendothelialization, and it also promotes the development of atherosclerotic plaques in mice
- AIM2 may have an important role in the development and progression of atherosclerosis
- AIM2 seems be a therapeutic target that is worth exploring further, and future studies focusing on the effects of AIM2 activation as well as pharmacological inhibition may reveal promising new therapeutic concepts for the treatment of atherosclerosis

## Introduction

One of the most common causes of heart disease, atherosclerosis, has been characterized as a chronic inflammatory disease. It arises from a combination of many factors, such as inflammation of the blood vessels, impaired vasodilation, decreased blood flow, development of plaques, and cholesterol deposition due to an imbalance of lipids in the blood (1). Despite intensive research efforts, the critical contributors and exact pathological mechanisms responsible for this disease are still insufficiently understood. Initially, it was assumed that vascular inflammation in atherosclerotic plaques was simply a secondary by-product of plaque development. Today, we know that activation of innate and adaptive immune responses is essential to the genesis of atherosclerosis and a major driving force for lesion formation (2).

Cardiovascular disease is triggered by endothelial damage that results from mechanical, biological, and toxic stress. Apoptosis and necrosis of vascular cells can lead to the activation of the innate immune system owing to the release of proteins, small molecules, and nucleic acids associated with cellular death. These signals are known as endogenous danger-associated molecular patterns (DAMP), which can activate pattern recognition receptors (PRR), leading to an innate immune response that helps clear the body of dead-cell debris and to neutralize potentially cytotoxic substances. PRR are specific innate immune receptors that have been closely linked to vascular inflammation and atherogenesis, which cause both damaging and attenuating effects. PRRs recognize DAMP of bacterial or viral origin and release proinflammatory cytokines to initiate anti-pathogenic signaling. PRRs can also be activated by endogenous ligands such as host DNA or RNA which can arise through cell death and lead to an immune response, although their role in the pathogenesis of atherosclerosis remains controversial, despite intensive research (3–6).

Absent in melanoma 2 (AIM2) belongs to the HIN-200 family of hematopoietic interferon (IFN)-inducible genes and is a PRR that is expressed in vascular cells that are specialized in detecting cytoplasmic dsDNA (7, 8). AIM2 can bind dsDNA directly through the HIN domain found at its carboxy-terminus, which then triggers assembly of the AIM2 inflammasome. Once assembled, the inflammasome can activate caspase-1 and cause cells to release mature IL-1β. Both caspase-1 activation and IL-1β release can only be triggered by the AIM2 inflammasome but not other members of the HIN-200 family (9, 10). Most previous studies have focused on the role of AIM2 in inflammation as a component of the innate immune system (7, 11, 12). Additionally, AIM2 has been shown to promote pyroptosis in tumor cells (13) and to be involved in the development of abdominal aortic aneurysms (14). Interestingly, Hakimi et al. found that endothelial cells (EC) respond to inflammatory signals by upregulating the expression of AIM2, indicating that there is a role for AIM2 in vascular inflammation (15). However, despite numerous studies focusing on AIM2, its role in vascular disease and atherosclerosis is not yet fully understood. To further investigate the biological effects of activating AIM2, we stimulated AIM2 *in vitro* as well as in acute and chronic vascular injury experiments *in vivo*. Finally, the role of AIM2 was studied in AIM2 knockout mice (AIM2^−/−^) in the acute vascular injury model.

## Methods

### Cell Culture

Human coronary artery endothelial cells (HCAEC) (Lonza, Basel, Switzerland) were cultured in endothelial cell growth medium (Promocell, Heidelberg, Germany) on 10 cm dishes at 37°C and with an atmospheric concentration of 5% CO_2_. Experiments were performed with cells from passages 5–8 when grown to 70–80% confluence. For stimulation studies, cells were incubated for 4 h with 25 ng poly dA:dT (Invivogen, Toulouse, France), 200 ng lipopolysaccharide (LPS), or phosphate-buffered saline (PBS) (as a control).

### Endothelial Scratch Assay

Endothelial scratch assays were performed as previously described (16, 17). Briefly, EC were grown to confluence and incubated with 100 ng/ml poly dA:dT or vehicle in cell culture medium for 18 h and subsequently scratched with a sterile pipette. The marked position was imaged every 2 h for 24 h. The remaining cell-free area was measured and a percentage of the initial scratched area was calculated.

### Apoptosis and Necrosis

To determine the amount of apoptosis and necrosis found in HCAEC, the Apoptotic/Necrotic/Healthy Cells Detection Kit (Promocell) was used according to the manufacturer's instructions. Briefly, EC were grown to confluence and then incubated with 100 ng poly dA:dT or vehicle in cell culture medium on a 24-well-plate for 24 h. Cells were stained green by using Annexin V with fluorescein (FITC; λabs/λem = 492/514 nm) to detect apoptotic and red with Ethidium Homodimer III (λabs/λem = 528/617 nm) to detect necrotic cells. To calculate the percentage of apoptotic and necrotic cells, cells were also stained blue by using Hoechst 33342 (λabs/λem = 350/461 nm).

### Real-Time Polymerase Chain Reaction (RT-PCR)

For analysis of gene expression levels in cultured HCAEC, cells were stimulated with 25 ng poly dA:dT for 4 h, lysed in Trizol (Invitrogen, Darmstadt, Germany), and the RNA was isolated with a peqGOLD RNA-Pure kit (peqLAB Biotechnology, Erlangen, Germany). The concentration and quality of the purified RNA were verified by using a spectrophotometer. Isolated total RNA (1 μg) was reverse transcribed by using the Omniscript RT Kit (Qiagen), according to the manufacturer's protocol. Single-stranded cDNA was then amplified by quantitative RT-PCR with the TaqMan system (ABI-7500 fast PCR System), with individual primers and SYBR-Green detection dye. Primers for human AIM2: sense 5′-GCGGTGTCTGCAGTGATGAA-3 ′, antisense 5′-GCAGATGCAGCAGGACTCAT-3′; 18s rRNA: forward 5′-GTAACCCGTTGAACCCCATT-3′; reverse 5′ -CCATCCAATCGGTAGTAGCG-3′. For quantification, mRNA expression was normalized to endogenous 18s rRNA.

### Animals

For this study, we used 8–12-week-old C57BL/6 wild-type (WT) mice (Charles River, Sulzfeld, Germany), 9–10-week-old ApoE^−/−^ mice on a C57BL/6 background (Charles River) and 8–12-week-old AIM2^−/−^ mice gene-targeted on a C57BL/6 background, who were kindly sponsored by Veit Hornung (Institute for Clinical Chemistry and Clinical Pharmacology, University Hospital of Bonn). The body weight of the mice varied from 22.5 to 28.5 g. All animals were maintained in a 22°C room with a 12 h light/dark cycle, and received food and drinking water *ad libitum*. To determine the concentration of poly dA:dT required for a proinflammatory response, WT mice were injected intravenously (i.v.) with 50, 75, or 100 μg poly dA:dT suspended in 200 μl PBS or vehicle (PBS) alone. Blood plasma from the mice was collected 2 h after injection. For the acute injury model, 50 μg poly dA:dT in 200 μl PBS per mouse was injected every 48 h for 7 days. For the analysis of atherosclerotic plaque development, ApoE^−/−^ mice received a cholesterol-rich diet that contained 21% fat, 19.5% casein, and 1.25% cholesterol (Ssniff, Soest, Germany) for a total of 8 weeks, and concomitantly were injected subcutaneously (s.c.) with either 50 μg poly dA:dT suspended in 200 μl PBS or vehicle every other day for 8 weeks. All tissue and blood samples were collected and processed immediately after mice were sacrificed. All mice were anesthetized using 2% isoflurane and intraperitoneal injection of fentanyl (0.05 mg/kg), midazolam (5 mg/kg), and medetomidine (0.5 mg/kg) and euthanized by cervical dislocation or exsanguination. The latter if carotid artery injury was performed. All animal experiments were performed in accordance with the Directive 2010/63/EU of the European Parliament. All animal experiments were approved by the local ethics committee of the University of Bonn (Permit Number: 84–2.04.2013.A197), supervised by the regulatory authority of the state of North Rhine-Westphalia and performed in compliance with German animal protection laws.

### Carotid Artery Injury

Carotid artery injury was performed as previously described (2) on day three of treatment with poly dA:dT, after the second i.v. poly dA:dT injection. All mice were anesthetized with intraperitoneal injections of 150 mg/kg body weight ketamine hydrochloride (Ketanest, Riemser, Greifswald, Germany) and 0.1 mg/kg body weight xylazine hydrochloride (Rompun 2%, Ceva, Duesseldorf, Germany). A small incision was made from the cranial apex of the sternum to just below the mandible. After careful preparation of an ~6 mm long segment proximal to the bifurcation, the common carotid artery was electrically denuded. A 4 mm long lesion was made by applying two serial 5 s bursts of 2 watts using a 2 mm wide forceps. The skin was then sutured, and the mice were allowed to recover in individual cages before returning to their littermates. On day seven of the poly dA:dT-treatment, 50 μl Evan's blue solution (5%, Sigma) was injected i.v. and allowed to circulate for 2 min. The mice were then sacrificed and both of the common carotid arteries were fully excised. The arteries were rinsed in a 0.9% sodium chloride solution, and the residual connective tissue was carefully removed. Images were taken, and the total lesion area (4 mm) along with the remaining denuded area (stained blue) were measured by using AxioVision version 4.8.2 software (Zeiss, Oberkichen, Germany). Reendothelialization was determined by calculating the remaining denuded area.

### Aortic Ring Preparations and Tension Recording

Vasodilation and vasoconstriction of isolated aortic ring segments were determined in organ baths filled with oxygenated modified Tyrode buffer (37°C), as previously described (2). Adventitial tissue was carefully removed, and 3 mm segments of the thoracic aorta were investigated. A resting tension of 10 mN was maintained throughout the experiment. Drugs were added in increasing concentrations in order to obtain cumulative concentration-response curves: KCl 20 and 40 mmol/l, phenylephrine 1 nmol/l−10 μmol/l, carbachol 10 nmol/l−100 μmol/l (for assessment of endothelium-dependent vasodilation after precontraction with phenylephrine) and nitroglycerin 1 nmol/l−10 μmol/l (for assessment of endothelium-independent vasodilation after precontraction with phenylephrine). The drug concentration was increased when vasoconstriction or vasorelaxation was completed. Drugs were washed out by Tyrode buffer before the next substance was added.

### Measurement of ROS

The release of superoxide in intact aortic segments was measured by L-012 chemiluminescence, as previously described (2, 18). Aortas were carefully excised and placed in chilled, modified Krebs-HEPES buffer (pH 7.4; in mmol/L: NaCl 99.01, KCl 4.69, CaCl_2_ 1.87, MgSO_4_ 1.20, Na-HEPES 20.0, K_2_HPO_4_ 1.03, NaHCO_3_ 25.0, and D-(+)-glucose 11.1). The connective tissue was removed and the aortas were cut into 2 mm segments. Chemiluminescence of the aortic segments was assessed in scintillation vials containing Krebs-HEPES buffer with 100 μmol/L L-012 over 10 min in a scintillation counter (Lumat LB 9501, Berthold, Bad Wildbad, Germany) in 1 min intervals. The vessel segments were then dried to determine their dry weight. ROS release was calculated as relative chemiluminescence per mg of aortic tissue as a percentage of the control.

### Cytokine Quantification

The concentration of interleukin-1β (IL-1β), interleukin-6 (IL-6), interleukin-18 (IL-18), interleukin-10 (IL-10), and tumor necrosis factor-α (TNF-α) were determined in the plasma of poly dA:dT-stimulated WT mice by ELISA. Commercially available ELISA kits were used according to the manufacturer's protocols (Qiagen, Hilden, Germany).

### Flow Cytometry

Murine blood samples were analyzed as previously described (19). Following lysis of the red blood cells, the viable lymphocyte population was analyzed for sca-1 (sca-1-FITC, Becton Dickinson, Franklin Lakes, USA), flk-1 (flk-1-PE, Becton Dickinson), Annexin V (Becton Dickinson), and CD 31 (Becton Dickinson). Isotype identical antibodies and unstained samples served as controls in each experiment (Becton Dickinson). Cell fluorescence was measured immediately after staining using fluorescence-activated cell sorting (FACSCalibur, Becton Dickinson). Data were analyzed using CellQuest software (Becton Dickinson). The units for each of the measured components were the number of specific events obtained after measuring 50,000 events in a pre-specified lymphocyte gate during FACS analysis.

### Histological and Immunohistochemical Analysis of Atherosclerotic Plaques

For histological analysis of atherosclerotic plaques, hearts with ascending aortas were embedded in Tissue-Tek (Miles, Elkhart, USA), snap frozen, and stored at −80°C. Samples were sectioned (6 μm) with a Leica cryostat, starting at the apex and progressing through the aortic valve area into the ascending aorta, and placed on poly-L-lysine coated slides. For the detection of atherosclerotic lesions and macrophage accumulation, the cryosections were fixed with 3.7% formaldehyde for 1 h, rinsed with deionized water, stained with oil red O working solution (0.5%) for 30 min, and rinsed again. Hematoxylin eosin staining was performed according to standard protocols. For immunohistochemical analysis of macrophages, slides containing aortic cryosections were incubated with acetone for 30 min at −20°C. After washing with PBS, the slides were preincubated with 10% normal goat serum (NGS, Sigma, St. Louis, USA) for 30 min at room temperature (RT). The primary antibody MOMA-2 (Acris, Herford, Germany) was diluted in 1% NGS and applied for 1 h at RT, then at 4°C overnight. 0.1 M Tris-buffer slides were incubated with an alkaline phosphatase-conjugated secondary antibody (Sigma) for 1 h at RT. FastRed (Sigma) was used as the chromogenic substrate for the alkaline phosphatase reaction. Nuclei were counterstained with hematoxylin (blue). Isotype-specific antibodies were used as negative controls. Tissue sections were washed and mounted with Aquatex mounting medium (Sigma) for light microscopic analysis. For quantification of the formation of atherosclerotic plaques, the lipid-staining area and total area of the serial histological sections were measured. Atherosclerotic data are expressed as the lipid-staining area as a percentage of the total surface area. The investigators who performed the histological analyses were blinded to the treatment of the respective animal group. All sections were examined under a Zeiss Axiovert 200 M microscope using AxioVision version 4.8.2 software.

### Statistical Analysis

Statistical analysis was carried out using GraphPad Prism® (version 7.0, GraphPad Software Inc). Kolmogorov-Smirnov test and D'Agostino-Pearson omnibus test were used to test normality of data distribution. In addition to normality, also equal variance was tested. Normally distributed data was tested using the unpaired *t*-test, non-normally distributed data using the Mann-Whitney *U*-test. Repeated measurements were analyzed using paired *t*-test or Wilcoxon matched pairs test, respectively. Data are presented as mean ± standard deviation (SD) and mean difference with 95% confidence intervals (CI). *P* < 0.05 were considered significant.

## Results

**Figure 1:**
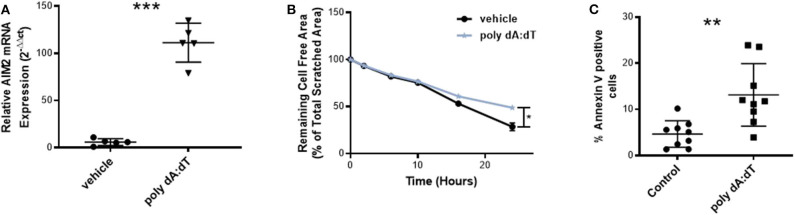
AIM2 stimulation *in vitro*. **(A)** AIM2 mRNA expression in human coronary artery endothelial cells (HCAEC) was significantly induced after 4 h of incubation with 25 ng poly dA:dT in comparison to vehicle treatment, each *n* = 5. **(B)** Stimulation of HCAEC with 100 ng poly dA:dT for 18 h significantly impaired reendothelialization in an *in vitro* scratch assay (*n* = 10). The remaining cell-free area as a percentage of the total scratched area is shown, which represents the amount of reendothelialization after 24 h. The reduced reendothelialization can be mainly attributed to a significant increase in apoptosis of HCAEC upon AIM2 stimulation. **(C)** Annexin V-positive cells as a percentage of the total cells are shown (*n* = 9). Kolmogorov-Smirnov test and D'Agostino-Pearson omnibus test were used to test normality of data distribution. Normally distributed data was tested using the unpaired *t*-test, non-normally distributed data using the Mann-Whitney *U*-test. Mean values ± SD are shown, *p* < 0.05 were considered as significant; ns, non-significant. * < 0.05, ** < 0.01, *** < 0.001.

To investigate the role of AIM2 activation in vascular biology, we first stimulated HCAEC with the AIM2-ligand, poly dA:dT. This treatment of endothelial cells significantly induced the expression of AIM2 mRNA, as measured by quantitative Real-time polymerase chain reaction (RT-PCR) (Figure 1A) and stimulation with 100 ng/ml poly dA:dT over 18 h significantly impaired reendothelialization compared to vehicle in an *in vitro* scratch assay (Figure 1B). The reduction in reendothelialization could be mainly attributed to a significant increase in apoptosis of HCAEC upon AIM2 stimulation (Figure 1C).

**Figure 2:**
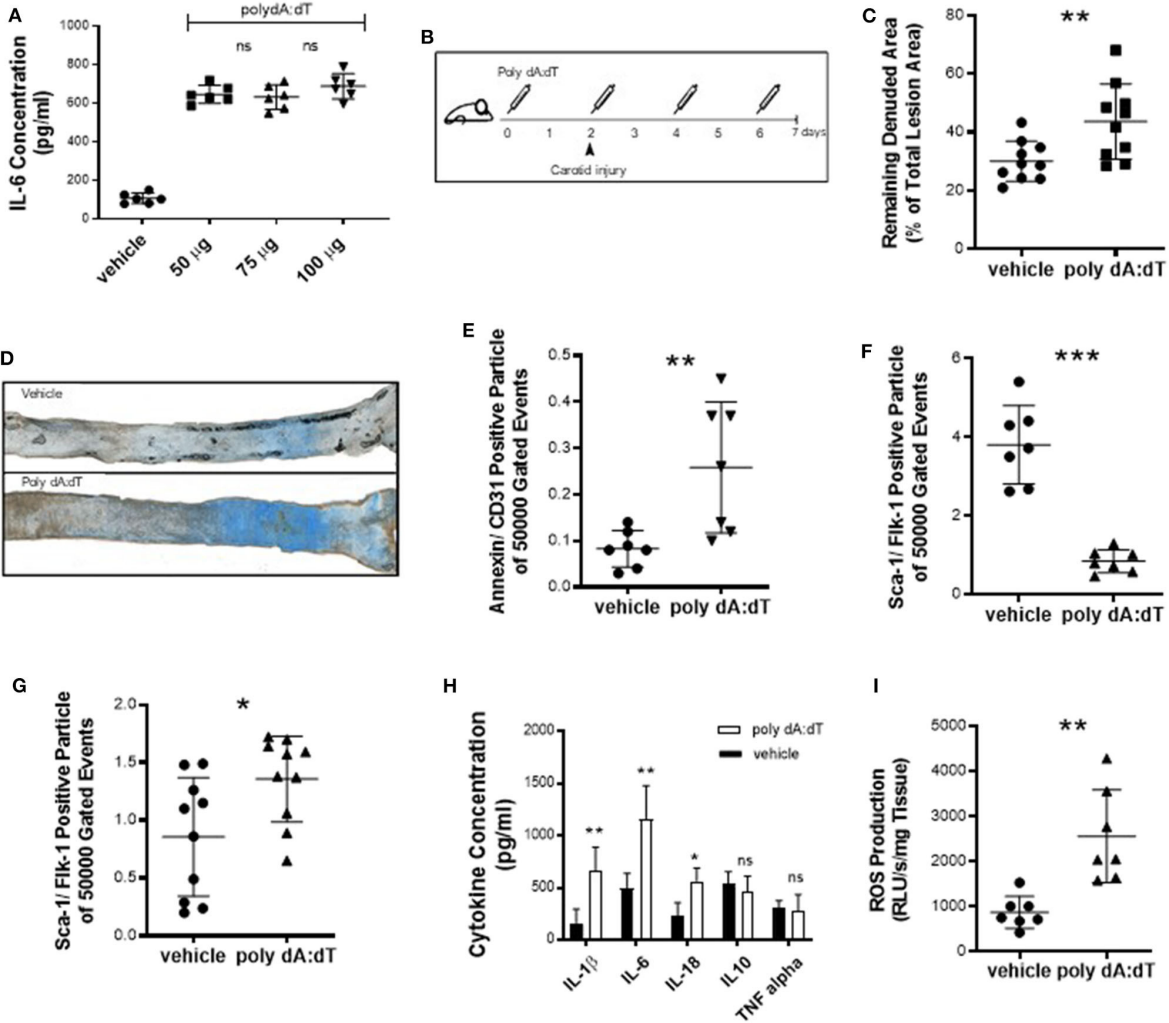
Acute vascular injury. **(A)** To determine the optimal dose of poly dA:dT, we injected WT mice (*n* = 6) with 50–100 μg poly dA:dT and measured the plasma IL-6 concentration. **(B)** WT mice were subjected to an electric denudation of the left common carotid artery and the reendothelialization was quantified 5 days after surgery. 50 μg poly dA:dT or vehicle (PBS) was injected i.v. every 48 h starting 2 days prior to the carotid injury. **(C)** Vascular reendothelialization was significantly impaired in AIM2-stimulated mice compared to vehicle controls, each *n* = 10. Remaining denuded area is shown as a percentage of the total lesion area. **(D)** Representative images of carotid arteries are shown with blue area indicating the remaining denuded area. **(E)** The number of EMP, defined as particles positively labeled for CD31 and Annexin V, was significantly elevated in poly dA:dT-treated mice (*n* = 7). **(F)** AIM2 stimulation nearly depleted the bone marrow of EPC, i.e., cells that stain positive for sca-1 and flk-1 (*n* = 7) (bone marrow) and **(G)** led to a significant increase of circulating sca-1/flk-1-positive cells (*n* = 10) (plasma). **(H)** Stimulation of AIM2 induced a systemic inflammatory response, measured by plasma levels of IL-1β, IL-6, and IL-18, each *n* = 9. **(I)** Production of ROS in the thoracic aorta was augmented in AIM2-stimulated mice compared to vehicle controls (*n* = 7). Kolmogorov-Smirnov test and D'Agostino-Pearson omnibus test were used to test normality of data distribution. Normally distributed data was tested using the unpaired *t*-test, non-normally distributed data using the Mann-Whitney *U*-test. Mean values ± SD are shown, *p* < 0.05 were considered as significant; ns, non-significant. * < 0.05, ** < 0.01, *** < 0.001.

Because reendothelialization and endothelial cell apoptosis have been associated with atherogenesis (20), we next sought to investigate the effects of systemic AIM2 stimulation *in vivo*. To induce a pro-inflammatory AIM2-mediated response, we injected WT mice with 50 μg poly dA:dT and measured the plasma concentration of IL-6 (Figure 2A). Vascular health depends on the ability of the endothelium to regenerate itself. This means that sites that undergo continual endothelial damage eventually lead to the development of vascular damage. An acute vascular injury model was used to study reendothelialization. WT mice were subjected to electric denudation of the left common carotid artery and reendothelialization was quantified 5 days after surgery. Poly dA:dT or vehicle was injected intravenously (i.v.) every 48 h, starting 2 days prior to the carotid injury (Figure 2B). Vascular reendothelialization was significantly impaired in AIM2-stimulated mice compared to vehicle controls (Figure 2C, quantification; Figure 2D, representative images). Reendothelialization in the present assay is dependent on two major factors, namely, total endothelial damage and the regenerative capacity of the endothelial cells. We therefore quantified the number of endothelial microparticles (EMP), as a marker of endothelial damage (21, 22). This number was significantly elevated in poly dA:dT-treated mice (Figure 2E). Additionally, AIM2 stimulation nearly depleted the bone marrow of sca-1/flk-1-positive cells (Figure 2F) while a significant increase was noted in circulating sca-1/flk-1-positive cells (Figure 2G). As expected, stimulation of AIM2 induced a systemic inflammatory response, measured by plasma levels of IL-1β, IL-6, and IL-18, whereas no significant difference was found for TNF-α and the anti-inflammatory cytokine IL-10 (Figure 2H). Finally, the production of ROS in the thoracic aorta (a source of oxidative damage (23)) was augmented in AIM2-stimulated mice compared to vehicle controls (Figure 2I).

**Figure 3:**
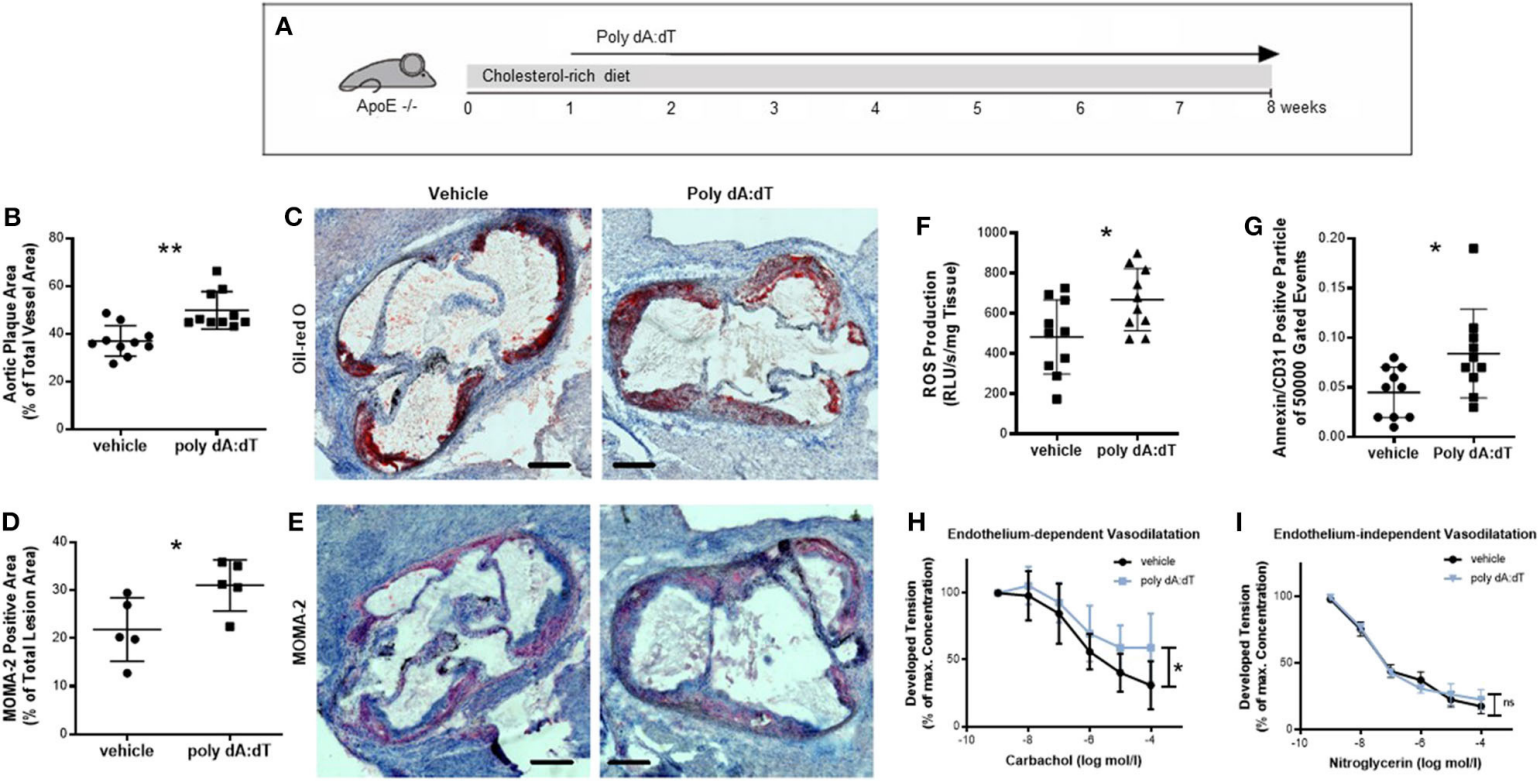
Chronic vascular injury. **(A)** ApoE^−/−^ mice received a high-fat, cholesterol-rich diet for a total of 8 weeks and were concomitantly injected s.c. with either 50 μg poly dA:dT or vehicle three times per week. **(B)** Poly dA:dT-treated ApoE^−/−^ mice developed significantly larger atherosclerotic plaques compared to control mice, measured as the aortic plaque area as a percentage of the total vessel area, each *n* = 10. **(C)** Representative oil red O stains of the aorta from vehicle- and poly dA:dT-treated ApoE^−/−^mice are shown. **(D)** Immunohistochemical staining with MOMA-2 showed a significant increase in macrophage infiltration (*n* = 5). **(E)** Representative MOMA-2 stains of the aorta from vehicle- and poly dA:dT-treated ApoE^−/−^mice are shown. **(F)** Poly dA:dT-stimulated mice showed a significant increase in vascular ROS formation compared to control animals, each *n* = 10. **(G)** The number of CD31/Annexin V-positive cells was significantly elevated in poly dA:dT-treated mice (*n* = 10). **(H)** AIM2 stimulation was associated with significantly impaired endothelium-dependent vasodilation, measured as the developed tension as a percentage of maximum contraction (*n* = 6). **(I)** Whereas, endothelium-independent vasodilation was not affected (*n* = 6). Kolmogorov-Smirnov test and D'Agostino-Pearson omnibus test were used to test normality of data distribution. Normally distributed data was tested using the unpaired *t*-test, non-normally distributed data using the Mann-Whitney *U*-test. Mean values ± SD are shown, *p* < 0.05 were considered as significant; ns, non-significant. * < 0.05, ** < 0.01.

We next investigated whether stimulation of AIM2 affects vascular biology in a chronic injury model. For this experiment, ApoE^−/−^ mice received a high-fat, cholesterol-rich diet for a total of 8 weeks and were concomitantly injected s.c. with either 50 μg poly dA:dT or vehicle three times per week (Figure 3A). Mice subjected to repetitive poly dA:dT injections showed no external signs of side effects; their body weight and food and water intake were indistinguishable from control mice (Supplementary Table 1). Poly dA:dT-treated ApoE^−/−^ mice developed atherosclerotic plaques that were significantly larger than those of the control mice (Figure 3B, quantitative; Figure 3C, representative image). Interestingly, the composition of the plaques was altered in AIM2-stimulated mice. Immunohistochemical staining for MOMA-2 (an antibody that stains macrophages) showed a significant increase in macrophage infiltration in poly dA:dT-treated animals compared to vehicle-treated controls (Figure 3D, quantification; Figure 3E representative image). Macrophages contribute to atherosclerotic plaque development in part by the production of ROS (24, 25). Therefore, we measured ROS production in the aortic segments of these mice. Mice treated with poly dA:dT had increased vascular ROS formation (Figure 3F), one of the main mediators of vascular dysfunction (26), and the number of EMP was significantly elevated compared to the control animals (Figure 3G). Finally, mice treated with vehicle alone displayed normal endothelium-dependent vasodilation in organ chamber experiments, whereas AIM2 stimulation in mice significantly impaired their endothelial function (Figure 3H). Endothelium-independent vasodilation (Figure 3I) and KCl- and phenylephrine-induced vasoconstriction (data not shown) were not affected by poly dA:dT treatment.

**Figure 4:**
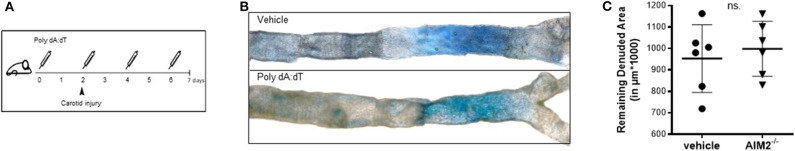
Acute vascular injury in AIM2^−/−^ mice. **(A)** AIM2^−/−^ mice were subjected to an electric denudation of the left common carotid artery and the reendothelialization was quantified 5 days after surgery. 50 μg poly dA:dT or vehicle (PBS), each *n* = 6, was injected i.v. every 48 h starting 2 days prior to the carotid injury. **(B)** Representative images of carotid arteries are shown with blue area indicating the remaining denuded area. **(C)** Treatment with poly dA:dT did not impair vascular reendothelialization in AIM2^−/−^ mice compared to vehicle controls, each *n* = 6. Remaining denuded area is shown. Kolmogorov-Smirnov test and D'Agostino-Pearson omnibus test were used to test normality of data distribution. Normally distributed data was tested using the unpaired *t*-test, non-normally distributed data using the Mann-Whitney *U*-test. Mean values ± SD are shown, *p* < 0.05 were considered as significant; ns, non-significant.

Finally, we investigated AIM2 knockout mice in our acute vascular injury model. AIM2^−/−^mice were subjected to electric denudation of the left common carotid artery and reendothelialization was quantified 5 days after surgery. Poly dA:dT or vehicle was injected intravenously (i.v.) every 48 h, starting 2 days prior to the carotid injury (Figure 4A). Here, treatment with poly dA:dT did not impair vascular reendothelialization in AIM2^−/−^ mice compared to vehicle controls (Figure 4B, representative images; Figure 4C, quantification).

## Discussion

**Figure 5:**
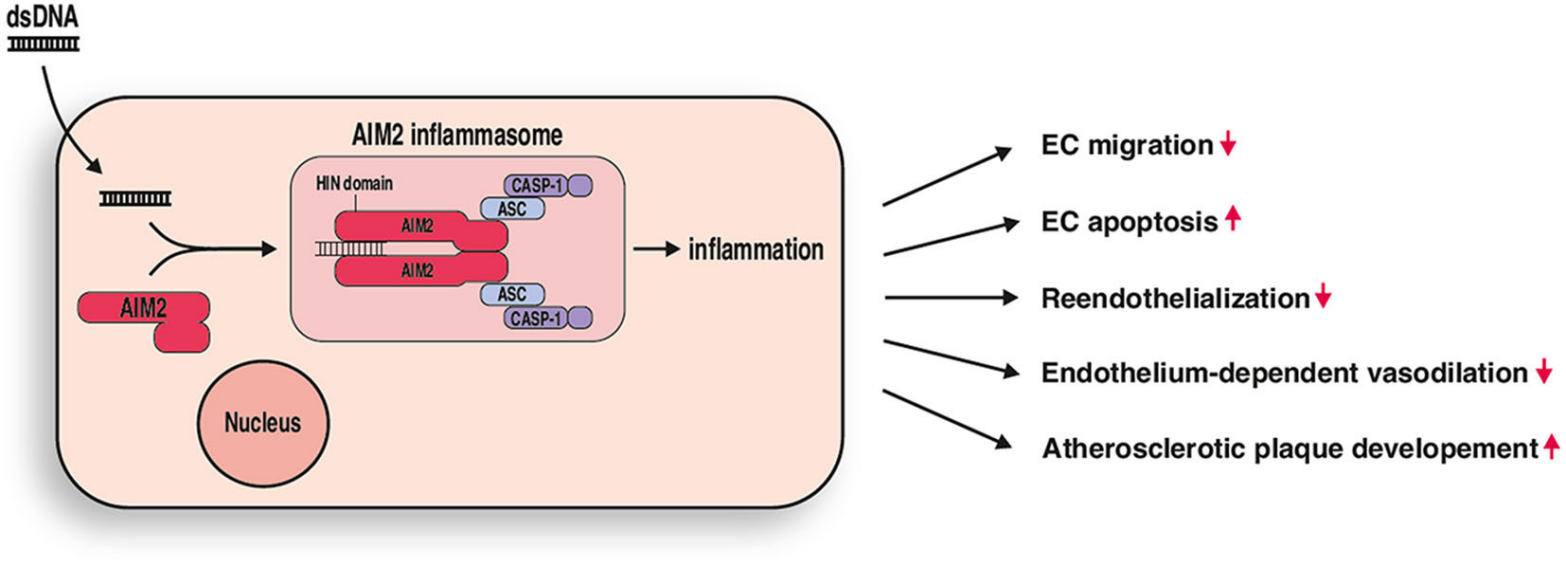
AIM2 mediated vascular inflammation. DsDNA stimulation leads to formation of an AIM2 inflammasome in endothelial cells. Resulting inflammation causes impaired endothelial cell (EC) migration and increased apoptosis *in vitro* and impaired reendothelization, endothelium-dependent vasodilation, and increased atherosclerotic plaque development *in vivo*.

Innate immunity with its many PRR is undoubtedly linked to atherosclerosis, a chronic inflammatory disease, which is influenced by multifactorial mechanisms at all stages of the disease (27). The central role of PRR in host defense is to detect pathogenic microorganisms, such as bacterial or viral molecules, and initiate an adequate immune response. However, several DAMPs can also occur endogenously and seem to play a major role in vessel inflammation and during monocyte recruitment to atherosclerotic lesions. Cell death, which is common in atherosclerotic lesions, leads to the release of intracellular material into the intercellular space, where it can be recognized by PRRs on resident or infiltrated cells. Multiple studies have investigated the role of the TLR3 inflammasome in this context and could demonstrate a central role during atherogenesis (28). For several of the other PRRs there remains controversy over whether they have pro- or antiatherosclerotic effects (24, 29), which illustrates the complexity of the innate immune system. For example, while TLR2 and TLR4 are considered to have pro-atherosclerotic effects (2, 30, 31), the presence of TLR7 and TLR9 in mice has been associated with a reduction in atherosclerotic lesions (19, 28, 32). The role of AIM2 in vascular disease and atherosclerosis remains insufficiently understood. Double-stranded DNA, which is inevitably a component of released intracellular content can activate AIM2 and lead to the assembly of an AIM2 inflammasome (33, 34) (Figure 5). To further investigate the biological effects of activating AIM2, we stimulated AIM2 *in vitro* as well as in acute and chronic vascular injury experiments *in vivo*.

AIM2 is expressed in EC of the intima and vasa vasorum of normal carotid arteries, as well as in aortas (15). Hakimi et al. found that inflammatory signals cause EC to respond by upregulating the expression of AIM2, which shows that there is a role for AIM2 in vascular inflammation, especially around the necrotic core of atherosclerotic carotid lesions and in the vasa vasorum neovasculature of aortic aneurysms. Furthermore, treatment of cultured primary human aortic endothelial cells and primary human aortic smooth muscle cells with TNF-α, IFN-γ, or cytosolic DNA induced endogenous expression of AIM2, which implies that AIM2 is an important factor in vascular pathogenesis (15).

In accordance with these findings, we stimulated HCAEC with poly dA:dT, which induced apoptosis and impaired migration, suggesting a proatherogenic influence for AIM2. We also found that AIM2 stimulation impairs reendothelialization following acute vascular injury, which was combined with elevated levels of EMP, ROS and sca-1/flk-1-positive cells. And by investigating AIM2 knockout mice in our acute vascular injury model without detecting significant differences we could demonstrate, that impaired reendothelialization is indeed mediated by AIM2. Similar results have been reported previously and are thought to be a result of impaired progenitor function due to the proinflammatory response (23, 35). Furthermore, increased levels of IL-6, IL-18, and IL-1β were found in poly dA:dT-treated mice. Poly dA:dT is a dsDNA PRR agonist which can be recognized by a number of cytosolic dsDNA sensors, including AIM2, cyclic GMP-AMP synthase (cGAS), DDX41, DNA-dependent activator of IFN-regulatory factors (DAI), Gamma-interferon-inducible protein Ifi-16 (IFI16), and indirectly by retinoic acid inducible gene I (RIG-I), leading to the production of type I interferons (36–39). However, only binding of the carboxy-terminal HIN domain of AIM2 to dsDNA triggers the assembly of an AIM2 inflammasome, which then results in the activation of caspase-1, leading to the cleavage of pro-IL-1β to its active form (9, 10). Results of the recent CANTOS trial (Canakinumab Anti-Inflammatory Thrombosis Outcome Study) have highlighted the importance of IL-1β for atherosclerosis, because patients with a history of atherosclerotic disease that were treated with an antibody against IL-1β experienced a significantly lower risk of having another cardiovascular event (40). Overall, activation of AIM2 in mice induced a systemic inflammatory reaction and disturbed the regeneration of endothelial cells after injury. Chronic AIM2 activation in ApoE^−/−^ mice increased the development of plaques and caused the accumulation of macrophages in aortic plaques compared to vehicle-treated mice; this was accompanied by elevated levels of ROS. Taken together, our data suggest that activation of AIM2 works in a pro-atherosclerotic manner to promote the disruption of endothelial function, higher production of ROS in the vessels, and a decrease in reendothelialization.

In line with our results, Pan et al. recently demonstrated that AIM2 promotes the progression of atherosclerotic plaques in ApoE^−/−^ mice by increasing migration in vascular smooth muscle cells (VSMC) (41). By intravenous injection of a murine AIM2 lentivirus, shRNA-AIM2 lentivirus, and null lentivirus into ApoE^−/−^ mice, the authors could show that the area of aortic atherosclerotic lesions was larger and the number of VSMC increased with elevated levels of AIM2. Overexpression of AIM2 induced higher expression of matrix metallopeptidase 2, an *in vitro* analysis revealed elevated AIM2 expression that depended on the level of oxidized low-density lipoprotein (ox-LDL), and trans-well-migration assays showed AIM2-mediated migration in VSMC. Pan et al. demonstrated that stimulation of AIM2 significantly affects cell migration induced by high levels of glucose and the TGF-β (transforming growth factor-β)/SMAD (small mothers against decapentaplegic) signaling pathways in VSMC. In addition, it has already been shown that enhanced atherosclerotic plaque formation following AIM2 stimulation in ApoE^−/−^ mice is accompanied by increased numbers of terminal deoxynucleotidyl transferase dUTP nick end labeling (TUNEL)—positive (apoptotic) cells. In addition, the expression of AIM2 and gasdermin D (GSDMD) expression were found to correlate with the concentration of ox-LDL in *in vitro* experiments, thus the effects of ox-LDL may occur through increasing AIM2 expression, leading to increased NF-κB signaling (42). Furthermore, Pan et al. found that AIM2 mediates GSDMD activity through the action of ASC (apoptosis-associated speck-like protein containing a CARD) and the caspase-1 pathway in VSMC. Finally, the authors concluded that AIM2 promotes the activity the N-terminal domain of GSDMD (GSDMD-N), which is required for pyroptosis (43), thereby accelerating pyroptosis in VSMC and unmasking AIM2 as an active participant in atherosclerosis. Overall, the findings mentioned provide further explanation for the proatherogenic effects of AIM2 stimulation in ApoE^−/−^ mice found in our study.

Particularly noteworthy in this context is the recently published study by Paulin et al. that perfectly supports and complements our findings. In their study, the authors compared arteriosclerotic lesion development in AIM2^−/−^ ApoE^−/−^ mice with ApoE^−/−^ littermates. Feeding the AIM2^−/−^ApoE^−/−^ mice a high-fat diet for 4 months caused a higher prevalence of intimal smooth muscle cells, which also led to a reduction in TUNEL staining. While the atherosclerotic lesions showed no difference in size, there was a larger amount of collagen, the fibrous caps were thicker, and there was a reduction of the area of the necrotic core. In general, the AIM2^−/−^ApoE^−/−^ mice from this study showed clear histopathological signs of having more stable lesions (33). Furthermore, the authors treated hypercholesteremic ApoE^−/−^ mice with a synthetic oligonucleotide A151 (or vehicle as a control) that acts as an antagonist for AIM2, during the last 8 weeks of a 4-month high-fat diet. Such pharmacological inhibition of AIM2 led to a significant expansion of smooth muscle cells in the lesions, reduced smooth muscle cell death, stimulated collagen deposition in the lesions, caused a thickening of the fibrous cap, and reduced the size of the necrotic core, in line with lesion characteristics of AIM2^−/−^ ApoE^−/−^ mice. In addition to these phenotypic responses, inhibition of AIM2 also caused the histopathological traits of the lesion to appear more stable as well as reducing IL-1β and IL-18 levels within the atherosclerotic lesions (33).

Herein, we have reported that AIM2 has an important role in the development and progression of atherosclerosis (Figure 5). It seems to be a promising therapeutic target that is worth exploring further, and future studies focusing on the effects of AIM2 activation as well as its pharmacological inhibition may reveal promising new therapeutic concepts for the treatment of atherosclerosis.

## Conclusion

In conclusion, we found that activation of AIM2 induced apoptosis and impaired migration in HCAECs. By using a mouse model of acute vascular injury, we determined that stimulating AIM2 systemically causes a decrease in reendothelialization as well as an increase in proinflammatory cytokine production and higher numbers of EMP and sca-1/flk-1-positive cells in the circulation. In particular, an increase in the development of atherosclerotic plaques and the overall concentration of ROS in aortic tissue was seen when ApoE^−/−^ mice were continuously treated with poly dA:dT. Overall, our data suggest that AIM2 is an active participant in atherosclerosis and underline the importance of further studies focused on the effects of AIM2 activation and inhibition for possible therapeutic targeting.

## Data Availability Statement

All datasets generated for this study are included in the article/Supplementary Material.

## Ethics Statement

All animal experiments were performed in accordance with the Directive 2010/63/EU of the European Parliament, approved by the local ethics committee, supervised by the regulatory authority of the state of North Rhine-Westphalia, and performed in compliance with German animal protection laws.

## Author Contributions

EL, PG, and AK designed the study, interpreted data, and wrote the manuscript. CL, AF, SN, and AZ performed laboratory measurements and analyzed data. GN and SZ designed the study, interpreted data, and critically revised the manuscript. FJ, CS, and SM critically revised the manuscript. All authors contributed to the article and approved the submitted version.

## Conflict of Interest

The authors declare that the research was conducted in the absence of any commercial or financial relationships that could be construed as a potential conflict of interest.

## Abbreviations

AIM2: absent in melanoma 2
ApoE: apolipoprotein E
DAMP: danger-associated molecular pattern
dsDNA: double-stranded deoxyribonucleic acid
EMP: endothelial microparticles
EPC: endothelial progenitor cells
EC: endothelial cells
FACS: fluorescence-activated cell sorting
GSDMD: gasdermin D
HCAEC: human coronary artery endothelial cells
IL-1β: interleukin-1β
IL-6: interleukin-6
IL-10: interleukin-10
IL-18: interleukin-18
LPS: lipopolysaccharide
NGS: normal goat serum
ox-LDL: oxidized low-density lipoprotein
PBS: phosphate buffered saline
Poly (dA:dT): poly(deoxyadenylic-deoxythymidylic) acid sodium salt
PRR: pattern recognition receptor
ROS: reactive oxygen species
RT: room temperature
TNF-α: tumor necrosis factor-α
TUNEL: terminal deoxynucleotidyl transferase dUTP nick end labeling
VSMC: vascular smooth muscle cell
WT: wild type.
