# Supplementary figures and images for: AIM2 Stimulation Impairs Reendothelialization and Promotes the Development of Atherosclerosis in Mice
Source: Front Cardiovasc Med. 2020 Nov 11;7:582482. doi: 10.3389/fcvm.2020.582482 (PMC7685997; doi:10.3389/fcvm.2020.582482)

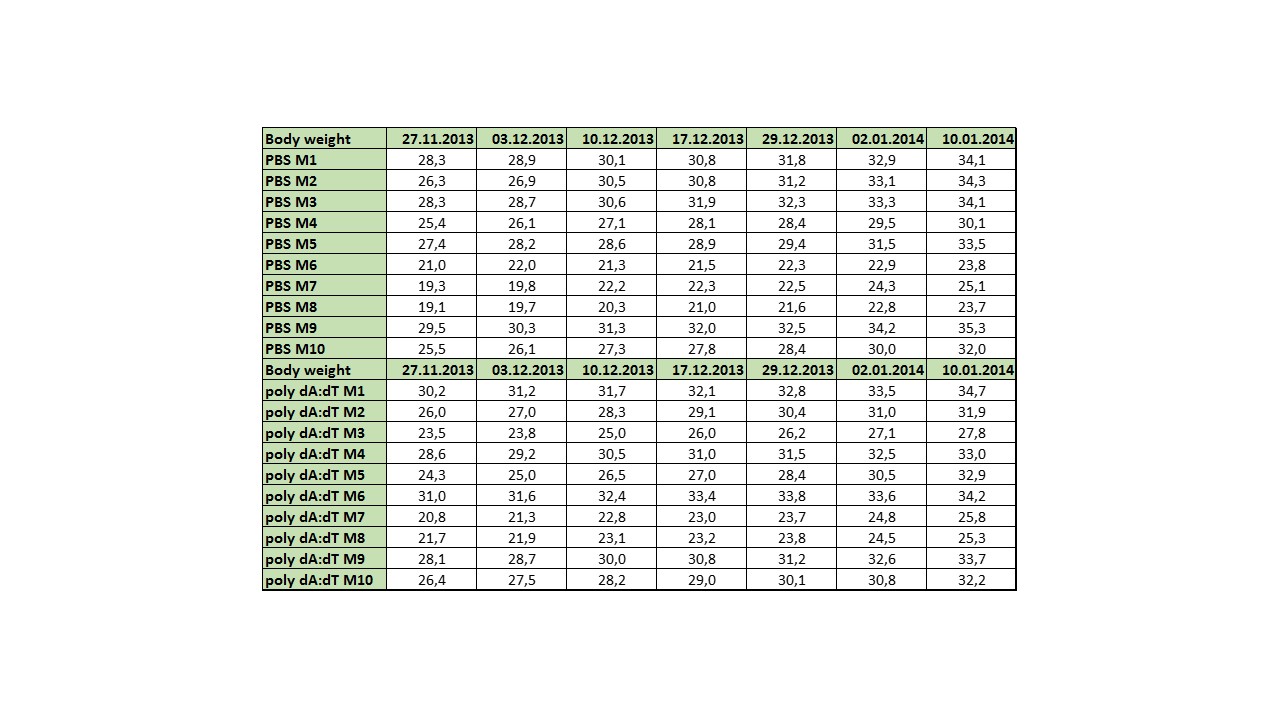

Supplement: Supplementary file 1 [file Image_1.jpg]
